# Dielectric-dependent electron transfer behaviour of cobalt hexacyanides in a solid solution of sodium chloride

**DOI:** 10.1039/c5sc02153g

**Published:** 2015-07-21

**Authors:** Di Huang, Yiliang Zhu, Ya-Qiong Su, Jie Zhang, Lianhuan Han, De-Yin Wu, Zhong-Qun Tian, Dongping Zhan

**Affiliations:** a State Key Laboratory of Physical Chemistry of Solid Surfaces , Department of Chemistry , College of Chemistry and Chemical Engineering , Xiamen University , 422 Siming South Road , Xiamen 361005 , China . Email: dpzhan@xmu.edu.cn

## Abstract

Here we emphasise the importance of the dielectric environment on the electron transfer behavior in interfacial electrochemical systems. Through doping cobalt hexacyanide (Co(CN)_6_^3–^) into single microcrystals of sodium chloride (NaCl), for the first time, we obtained the direct electrochemical behavior of Co(CN)_6_^3–^ which is hardly ever obtained in either aqueous or conventional nonaqueous solutions. DFT calculations elucidate that, as the Co(CN)_6_^3–^ anions occupy the lattice units of NaCl_6_^5–^ in the NaCl microcrystal, the redox energy barrier of Co(CN)_6_^3–/4–^ is decreased dramatically due to the low dielectric constant of NaCl. Meanwhile, the low-spin Co(CN)_6_^4–^ anions are stabilized in the lattices of the NaCl microcrystal. The results also show that the NaCl microcrystal is a potential solvent for solid-state electrochemistry at ambient temperature.

## Abstract 2

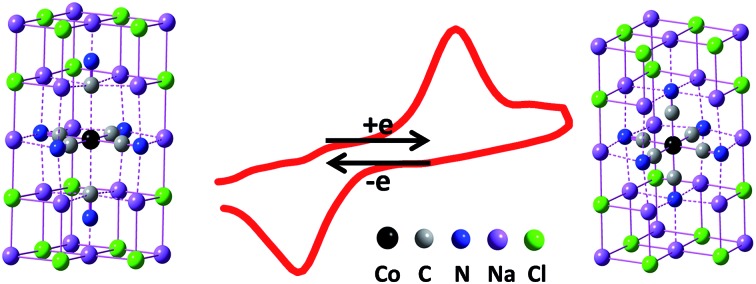
The electron transfer behavior of cobalt hexacyanide is obtained in a solid solution of sodium chloride due to the low dielectric environment.

## Introduction

The dielectric property of solvents, crucial in interfacial electron transfer reactions, has been paid little attention in the initiative design of electrochemical systems. In principle, the hexacyanides of transition metals have a good capability of electron transfer due to both the multivalent central cations and the homogeneous charge distribution. For example, potassium ferricyanide (K_3_Fe(CN)_6_), where the standard electrode potential of the Fe(CN)_6_^3–/4–^ couple is 0.361 V *versus* the normal hydrogen electrode (NHE) at 25 °C, is used widely as a classic redox couple in electrochemistry.^1^ The reversible electron transfer behavior underlies the determination of the effective area of the glassy carbon electrodes.^2^ However, despite the similar molecular structure of cobalt hexacyanide (Co(CN)_6_^3–^) to Fe(CN)_6_^3–^, redox behavior of Co(CN)_6_^3–^ in conventional aqueous or nonaqueous solutions has been seldom reported to date. Although theoretical calculation implies the possibility of the dielectric constant of the electrolyte solution to be decreased to 5, this indication has never been validated experimentally due to the lack of a qualified solvent.^3^ Since the dielectric constant of NaCl (*ε*: 6.0 F m^–1^) is very close to 5 F m^–1^, based on our previous research on solid solutions of NaCl microcrystals,^4^ we synthesize Co(CN)_6_^3–^ doped NaCl microcrystals and observed the direct electrochemical behavior of Co(CN)_6_^3–/4–^ in the dielectric environment of the NaCl microcrystal.

**Fig. 1:**
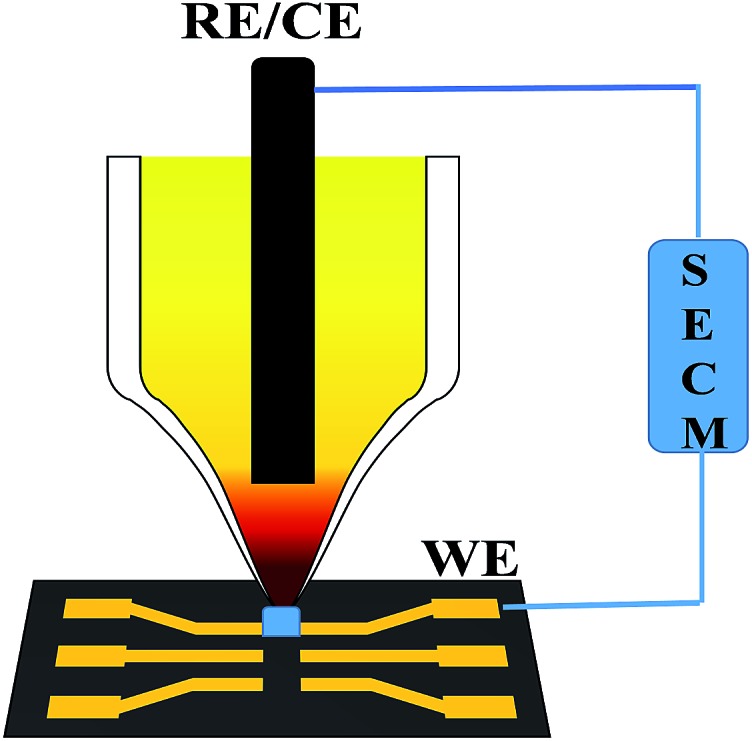
Schematic diagram of the assembly of the Co(CN)_6_^3–^/NaCl microcrystals into an electrochemical chip by SECCM: a microcapillary with a micrometer-sized orifice was employed as both the scanning tip and the electrochemical cell; the reference and counter electrodes were inserted in the microcapillary, and a pair of gold microelectrodes was used on the electrochemical microchip as the working electrodes.

We have developed a scanning electrochemical cell microscopy (SECCM) method to culture the NaCl solid-solution microcrystals, wherein the redox couples are doped as solute.^4^ Derived from scanning electrochemical microscopy, SECCM employs a micropipette with a micro- or nano-meter sized orifice as both the scanning tip and the electrochemical cell (Fig. 1);^5^ it has been proved as an effective technique in various research areas including in reaction kinetics,^6^ micro- or nano-patterning and imaging,5e,^7^ local corrosion or deposition,^8^ and also in catalyst screening.^9^ It was found that the Fe(CN)_6_^3–/4–^/NaCl solid-solution microcrystals have excellent solid-state redox behaviors in the absence of any liquid electrolyte, because the doped redox couple makes them electronic conductors while the crystal defects make them ionic conductors.4a,b Here, for the first time, we report the dielectric environment dependent, direct electron transfer behavior of Co(CN)_6_^3–/4–^ in NaCl microcrystals.

## Experimental section

### Chemicals, materials, and instruments

NaCl and Na_3_Co(CN)_6_ were of analytical grade or better (Sinopharm Co., China). All aqueous solutions were prepared with deionized water (18.2 MΩ, Milli-Q, Millipore Corp.). The borosilicate micropipetts (o.d., 1.2 mm; i.d., 0.8 mm) with an orifice of 3–10 μm diameter were prepared with a programmed laser puller PS-2000 (Sutter Co., USA) as reported previously.^4^,^15^ The Au and Pt thin-film coated glass slides were prepared through magnetron sputter plating (JC500-3/D, Chengdu Vacuum Equipment Co., China). The ITO glass slides are a kind gift from Prof. Bin Ren at Xiamen University. Before experiments, the slides were cleaned with acetone and deionized water several times and dried with pure nitrogen gas. A scanning electron microscope (SEM, Hitachi High-Technologies Co., Japan) was employed to obtain geometric topography and elementary analysis of the single microcrystals. Confocal Raman spectrum experiments were performed with a Renishaw inVia Raman microscope (Renishaw Co., British) to confirm the composition of microcrystalline solution. All electrochemical experiments were performed with the SECM workstation CHI920c (CHI Instrument Co., USA).

### Culture and assembly of the Na_3_Co(CN)_6_/NaCl microcrystals

We have developed the SECCM technique to culture and to assemble the Na_3_Co(CN)_6_/NaCl microcrystal.^4^ A micropipette with a micrometer sized orifice acts as both the scanning tip and the electrolytic cell. An Ag/AgCl wire was inserted into the micropipette as both the counter and reference electrodes. A conductive substrate, such as an ITO, Au, or Pt thin-film-coated glass slide, was the working electrode. With the help of the video camera, the micropipette was moved to be in contact with the conductive substrate. Since the tip and substrate were in contact with each other through a ∼picoliter or ∼femtoliter drop of electrolyte solution, the electrochemical reactions were confined in the small volume of the microdrop between the tip and the substrate. Actually, the spatial resolution of the SECCM depends on the size of the microdrop. When the tip was scanning, the whole electrochemical microsystem moved. Due to the evaporation of the water, the microcrystal can be obtained on the substrate. In order to form an electrochemical system, the microcrystal was assembled on the gap between a pair of gold microwires, which was deposited on a microchip made by lithography techniques.

### Theoretical calculations

The quantum-chemical calculations were carried out for geometry optimizations at the density functional theory (DFT) level with the hybrid functional B3LYP by using the Gaussian 09 package.^16^ For the C and N atoms, the basis set used was 6-311+G**.^17^ For the Co atom, the small core pseudopotential basis set LanL2DZ was adopted.^18^ To take the solvent effect into account, the Polarized Continuum Model (PCM) was used in all calculations.^19^ The dielectric constant *ε* is 78.3 F m^–1^ for water as well as 6.0 F m^–1^ for the NaCl solid-solution microcrystal. The Potential Energy Curves (PES) were fitted by optimizing and obtaining some special single point energies along the reaction coordinates.

## Results and discussion

As depicted in Fig. 1, the micropipette contacts with the conductive substrate through an electrolyte microdrop with a volume of pico or femto liter to construct the electrochemical microsystem. Water will evaporate since the microdrop is exposed to the atmosphere. From the Kelvin equation:^10^

1 *RT* ln(*P*/*P*_0_) = 2*γM*/*ρr*

**Fig. 2:**
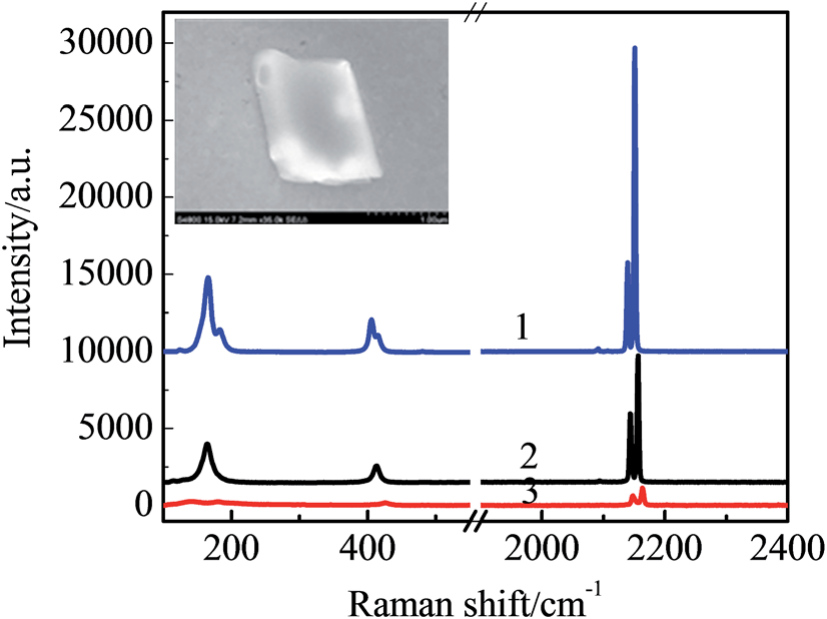
The confocal Raman spectra of the pure Na_3_Co(CN)_6_ crystals (curve 1), and the single Na_3_Co(CN)_6_/NaCl solid solution microcrystals with initial Na_3_Co(CN)_6_ concentration of 10 mM (curve 2) and 1 mM (curve 3); the concentration of the NaCl in the precursor solution is 50 mM. The inset is a SEM image of a single Na_3_Co(CN)_6_/NaCl microcrystal.

The smaller the *r* is, the higher the *P* is, and the faster the water evaporates, where *P* is the actual evaporation pressure, *P*_0_ the saturated evaporation pressure, *γ* the surface tension, *M* the molecular weight of the electrolyte, *ρ* the density of the electrolyte, *r* the radius of the microdrop, *R* the gas constant and *T* the absolute temperature. Cyclic voltammetry with a scanning rate of 50 mV s^–1^ and a scan range between 0 mV and 500 mV is performed to modulate the surface tension and, therefore, the process of water evaporation. In general, it takes a few cycles to form a well-shaped microcrystal (shown in the insert in Fig. 2). If the electrochemical modulations were not applied, it would take more time for the microcrystal growth to occur. Meanwhile, the shape of the microcrystals would become uncontrollable as reported previously.^4^

Since the lattice size of Na_3_Co(CN)_6_ (9.39 Å) is very close to that of NaCl (9.2 Å), this means that the Co(CN)_6_^3–^ units can take the place of the NaCl_6_^5–^ units and dope into the NaCl microcrystal to form a solid solution.^11^,^12^ As the valence of the Co^3+^ is higher than the Na^+^, to achieve electroneutrality, two Na^+^ vacancies are left in the neighboring NaCl_6_^5–^ units. That means the Na_3_Co(CN)_6_/NaCl solid solution is a Na^+^ ionic conductor. Due to the difference between the lattice sizes of the Na_3_Co(CN)_6_ and the NaCl (2.06%), to some extent, the microcrystals have the characteristics of twin crystals. The crystal defects, including both vacancies and interstitials, are essential to improve the ionic conductivity of the Na_3_Co(CN)_6_/NaCl solid-solution microcrystals.^13^ The component of the Co(CN)_6_^3–^ in the NaCl microcrystal is verified by the confocal Raman spectra (Fig. 2). The characteristic bands obtained from the microcrystals are in accordance with that of the pure Na_3_Co(CN)_6_ crystals. The bands at 116 cm^–1^ and 405 cm^–1^ are assigned to the Fe–C stretching while the bands at 2139 cm^–1^ and 2151 cm^–1^ are assigned to the C≡N stretching. The band strength is relative to the initial concentration of Na_3_Co(CN)_6_ in the precursor solution.

**Fig. 3:**
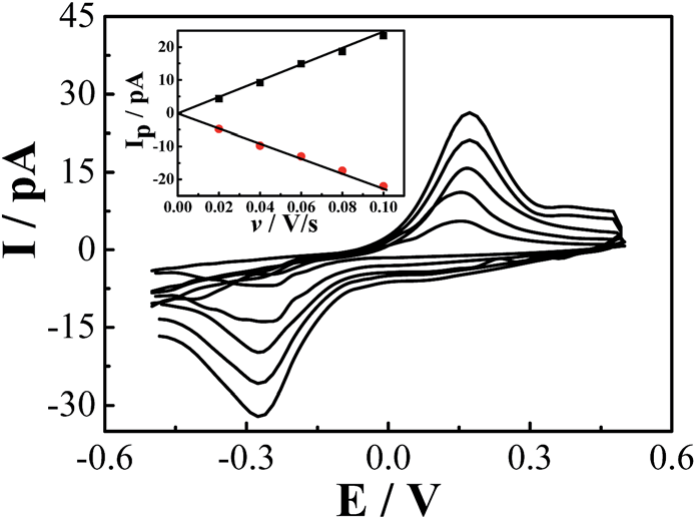
Cyclic voltammetric behavior of Co(CN)_6_^3–/4–^ in the NaCl microcrystals in the dry box. The precursor solution for the culturing of the NaCl microcrystals contained 10 mM Co(CN)_6_^3–^ and 50 mM NaCl. The insert shows the linear relationship between the peak current and the scanning rate.

A single Na_3_Co(CN)_6_/NaCl microcrystal is assembled into the gap between a pair of gold microwires on a microchip by SECCM to construct an all-in-solid electrochemical system as reported previously (Fig. 1).^4^ Well-defined voltammetric behaviors of Co(CN)_6_^3–^ in the NaCl microcrystal were obtained as shown in Fig. 3. The redox process can be formulated as:

2 Co(CN)_6_^3–^ + e ⇌ Co(CN)_6_^4–^

The difference of the peak potentials between the anodic and cathodic processes is rather big (∼450 mV), which indicates that the electron transfer reaction is irreversible and kinetically slow. In other words, both the anodic and the cathodic processes need a large overpotential. From the insert of Fig. 3, the peak current has a good linear relationship with the scanning rate. Because the redox units of Co(CN)_6_^3–^ are trapped in the lattices of the NaCl microcrystal, the electron transfer occurs through electron hopping or self-exchange between the neighbouring Co(CN)_6_^3–^ units. Meanwhile, to maintain the electroneutrality of the NaCl microcrystal, the local charges caused by the electron transfer are compensated by the diffusion and electromigration of the counterion Na^+^. Supposing that the electron transfer behavior is similar to the thin-layer voltammetry as analyzed before, the relationship between the peak current and the scanning rate for an irreversible process can be expressed as:4a,4b,^14^

3

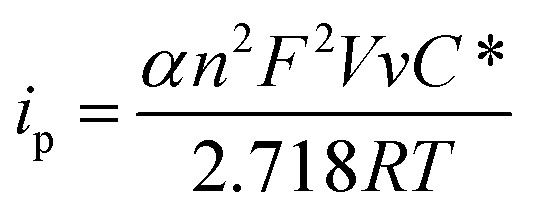

where *i*_p_ is the peak current, *α* the charge transfer coefficient, *n* the stoichiometric charge transfer number, *F* the Faraday constant, *V* the volume of the microcrystal, *v* the scanning rate, C* the bulk concentration of the reactant and *R* and *T* are the same as indicated in eqn (1). The dimensions of the single Na_3_Co(CN)_6_/NaCl microcrystals are about 2 μm × 2 μm × 2 μm. If *α* is 0.5, the apparent concentration of the Co(CN)_6_^3–^ in the NaCl microcrystals can be estimated as 4.3 × 10^–5^ mol cm^–3^.

**Fig. 4:**
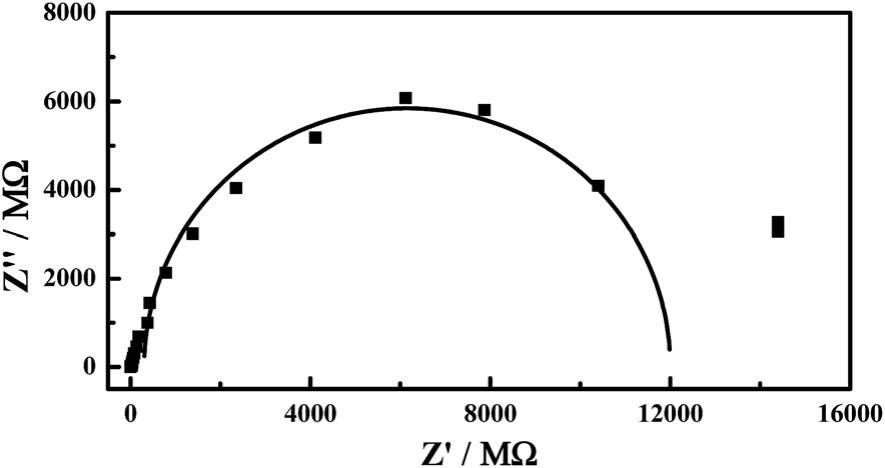
The electrochemical impedance spectroscopy of the redox couple of Co(CN)_6_^3–/4–^ in the NaCl microcrystals. The initial concentration of Co(CN)_6_^3–^ for the crystal culture is 10 mM.

Furthermore, electrochemical impedance spectroscopy (EIS) was performed to obtain the kinetic rate of the electron transfer (shown in Fig. 4). Considering that the redox process of Co(CN)_6_^3–^ is a simple one-electron transfer reaction as shown in eqn (2), in the range of high frequency, the following equation should be applied:^1^

4 (*Z*′ – *R*_u_ – *R*_et_/2)^2^ + *Z*′′^2^ = (*R*_et_/2)^2^

where *R*_u_ is the Ohm resistance of the single microcrystal, *R*_et_ is the electron transfer resistance and *Z*′ and *Z*′′ are the real and imaginary parts of the impedance, respectively. The electron transfer resistance (*R*_et_) is derived as 1.21 × 10^10^ Ω. By using the geometric parameters and the apparent concentration of the Co(CN)_6_^3–^ obtained by cyclic voltammetry, the kinetic rate of the electron transfer, *k*_et_, is calculated as 1.24 × 10^–5^ cm s^–1^. However, Fig. 4 can't provide the mass transfer information, *i.e.*, the diffusion of the counterion Na^+^ in the NaCl microcrystal. Our previous study shows that the apparent diffusion coefficient (*D*) of the Na^+^ in the Na_3_Fe(CN)_6_/NaCl microcrystals is 8.05 × 10^–8^ cm^2^ s^–1^.4*b* Considering the similarity of the Na_3_Co(CN)_6_/NaCl microcrystals, the mass transfer rate, *k*_mt_, is estimated as 4.0 × 10^–4^ cm s^–1^ if the thickness of diffusion layer (*δ*) is 2 μm (*k*_mt_ = *D*/*δ*). Note that the mass transfer rate is much higher than the electron transfer rate (*k*_mt_ > 20*k*_et_); it can be concluded that the rate-determining step (rds) of the redox reaction of Co(CN)_6_^3–^ in the NaCl microcrystal is the electron propagation in the NaCl microcrystals. The EIS results elucidate, in turn, the validity of the “irreversible” hypothesis in the discussion section of the cyclic voltammetry.

**Fig. 5:**
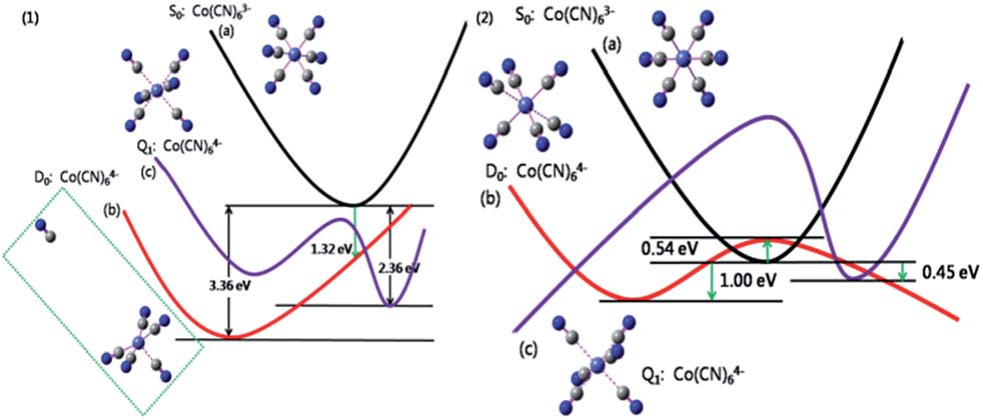
(1) Qualitative potential energy curves of (a) the Co(CN)_6_^3–^ anion (S_0_, black line), (b) the Co(CN)_6_^4–^ anion in the low-spin state (D_0_, red line), and (c) the Co(CN)_6_^4–^ anion in the high-spin state (Q_1_, purple line) at the PCM-B3LYP/6-311+G**/LANL2DZ level in an aqueous solution (*ε*: 78.3 F m^–1^). (2) Potential energy curves of (a) the Co(CN)_6_^3–^ anion (S_0_, black line), (b) the Co(CN)_6_^4–^ anion in the low-spin state (D_0_, red line), and (c) the Co(CN)_6_^4–^ anion in the high-spin state (Q_1_, purple line) at the PCM-B3LYP/6-311+G**/LANL2DZ level in a NaCl solid solution (*ε*: 6.0 F m^–1^).

It should be noted that this reaction can't be observed in conventional solvents even if a mercury electrode is employed to extend the potential window further into the negative.^3^ To elucidate the unique redox behavior of Co(CN)_6_^3–/4–^ in the NaCl microcrystal, theoretical calculations were performed using a density functional theory (DFT) method. Fig. 5 gives the potential energy curves (PEC) of the electrochemical reduction process of the Co(CN)_6_^3–^ anion in both an aqueous solution (*ε*: 78.3 F m^–1^) and a NaCl solid solution (*ε*: 6.0 F m^–1^). In aqueous solution, the predicted reorganization energy *λ* is 2.04 eV after accepting an electron to form the low-spin Co(CN)_6_^4–^ anion, which is instable and quickly decomposed into Co(CN)_5_^3–^ and CN^¬^ ions by the rupture of the Co–CN bond due to the introduction of an electron. Fig. 5(1b) gives the corresponding optimization structure, and also the distance between the dissociated CN^¬^ and Co(CN)_5_^3–^ anion, which is 10.57 Å. It is obvious that there is no bonding interaction between them. However, the high-spin Co(CN)_6_^4–^ anion is relatively stable and keeps the original geometric symmetry with a reorganization energy of 1.59 eV. It should be noted that the S_0_ and D_0_ PECs have no cross points along the electron transfer reaction coordinate and, if an electron is injected, the relaxation process of the Co(CN)_6_^3–^ anion would be irreversible with an enormous stabilization energy of 3.36 eV. In summary, in an aqueous solution, the electrochemical reduction product of the Co(CN)_6_^3–^ anion prefers to be the low-spin Co(CN)_5_^3–^ and the CN^¬^ ion, and the predicted reduction potential is about –0.98 V *vs.* NHE. Unfortunately, in an aqueous solution this redox behavior hasn't been observed experimentally.^3^

However, in the NaCl solid-solution microcrystal, the S_0_, D_0_ and Q_1_ PEC are intersectional. For the reduction of the Co(CN)_6_^3–^ anion, the reorganization energy is about 1.54 eV. The injected electron just climbs over a low energy barrier (<0.54 eV) to enter the D_0_ PEC and relaxes into the product, the low-spin Co(CN)_6_^4–^ anion, which is stabilized and keeps the original coordination number in the dielectric environment of the NaCl microcrystal. Meanwhile, the oxidation energy barrier of the reverse process is just between 1.00 and 1.54 eV. The low dielectric constant (*ε*: 6.0 F m^–1^) leads to a smaller stabilization energy of 1.00 eV and, consequently, an electrochemical redox process of the Co(CN)_6_^3–/4–^ couple. It can be concluded that the dielectric environment plays a vital role in not only the thermodynamic possibility but also the electrode kinetics of the electron transfer processes. The NaCl solid-solution microcrystal can provide a suitable dielectric environment for the hexacyanides of transition metals which are expected to have good electron transfer properties but, for some reason, aren't present in the conventional electrolyte solutions at ambient temperature.

## Conclusions

For the first time, we obtained the well-defined redox behavior of Co(CN)_6_^3–/4–^ in single NaCl microcrystals. The formation mechanism of the Co(CN)_6_^3–^/NaCl solid solution is confirmed by both crystallographic principles and a confocal Raman spectra experiment. The electrochemical investigations show that the electrode process is kinetically controlled, which is elucidated in the DFT calculations. DFT results show that the reactant, Co(CN)_6_^4–^, is stabilized and the active energy of the redox couple, Co(CN)_6_^3–/4–^, is lowered in the special dielectric environment of the NaCl microcrystals. This dielectric-dependent electron transfer behavior recalls the crucial role of the dielectric environment of the electrochemical system. A proper dielectric environment can present the electron transfer behavior expected in theory, but hardly ever obtained in experiments. Moreover, the NaCl solid solution is proved as a prospective solvent for solid-state electrochemistry in ambient temperature, which might have potential application in all-in-solid sensors or power sources.
